# Drug-induced musical hallucination

**DOI:** 10.3389/fphar.2024.1401237

**Published:** 2024-05-22

**Authors:** Brock Bakewell, Michael Johnson, Madison Lee, Elizabeth Tchernogorova, Jesse Taysom, Qing Zhong

**Affiliations:** Rocky Vista University College of Osteopathic Medicine, Ivins, UT, United States

**Keywords:** musical hallucination, drug side effect, mechanism, treatment, drugs-induced musical hallucination

## Abstract

Musical hallucination is a rare perceptual phenomenon wherein individuals hear music in the absence of external auditory stimuli. This phenomenon occurs across diverse medical conditions and can be triggered by some drugs. The underlying mechanism of drug-induced hallucination is unknown. This study explores drug-induced musical hallucination through a literature review, aiming to investigate its pathophysiology and potential treatment modalities. A literature search was conducted until January 2024 using databases PubMed, WorldCat, Google Scholar, and DOAJ, with keywords “drugs induced musical hallucination” or “drugs” combined with “musical hallucination.” The search yielded 24 articles which met inclusion criteria, encompassing 27 cases. The average patient age was 58.3 years, with 67.9% females. Prevalent conditions among cases included hearing impairments, psychiatric disorders, cancers, and neurodegenerative conditions. Common trigger drugs comprised antidepressants, opioids, anti-Parkinson drugs, ketamine, and voriconazole. Musical hallucination descriptions varied widely, and 6 patients reported concurrent visual hallucinations. The onset of symptoms ranged from 75 min to 240 days. Treatment strategies included termination of trigger drugs, dosage reduction, alteration of administration routes or formula, switching to similar drugs, or addition of antidepressants, sedatives, or atypical antipsychotic medications. Musical hallucinations completely disappeared in 24/27 (88.9%) patients but continued in 3/27 (11.1%) patients. The current study concludes that drug-induced musical hallucination may arise from altering neurotransmitter/receptor balance and intricate interactions between trigger drugs and underlying conditions.

## 1 Introduction

Musical hallucination is a perceptual phenomenon where an individual hears music or songs in the absence of an external auditory stimulus (Evers and Ellger, 2004; Golden and Josephs, 2015). It is classified under the broader category of auditory hallucinations. While all musical hallucination instances are considered auditory hallucinations, not all auditory hallucinations manifest as musical experiences. These musical hallucination episodes can range from simple tunes and melodies to complex orchestral compositions, spanning diverse musical genres (Golden and Josephs, 2015).

Some medical conditions could present musical hallucinations, including hypoacusis or hearing loss, psychiatric disorders, and brain structural/functional lesions (Prommer, 2005; Golden and Josephs, 2015). Although dopaminergic stimulation is hypothesized to contribute to auditory hallucination, for example, in schizophrenia, the precise mechanisms underlying musical hallucination remain unclear (Sivanesan et al., 2016).

Furthermore, some drugs were reported to cause musical hallucinations, including ketamine, opioids, tricyclic antidepressants, voriconazole, antiparkinson, and benzodiazepines (Prommer, 2005; Golden and Josephs, 2015). As aforementioned, dopamine overactivation might cause auditory hallucinations. Coincidently, opioids were found to increase extracellular dopamine concentration in a dose-dependent manner in the nucleus accumbens of rats (Tanda et al., 1997). Therefore, dopamine pathway dysregulation is implied to contribute to the occurrence of auditory hallucinations in opioid users (Sivanesan et al., 2016). However, no clinical trials investigating drug-induced musical hallucination were reported, the exact mechanism of drug-induced musical hallucination is unknown.

While musical hallucinations have been linked to many conditions or trigger drugs (Evers and Ellger, 2004; Cope and Baguley, 2009; Coebergh et al., 2015), there is a notable gap in reviews focusing specifically on drug-induced musical hallucinations. This study aims to fill these gaps, exploring especially drug-induced musical hallucination. By investigating the interactions between trigger drugs and underlying conditions, we seek to contribute valuable insights into pathophysiology and treatment which may guide future research in this intriguing field.

## 2 Methods

A literature search was conducted across four databases, PubMed, WorldCat, Google Scholar, and Directory of Open Access Journals (DOAJ), until January 2024. The search words included “drugs-induced musical hallucination” or “drugs” AND “musical hallucination.” The inclusion criteria encompassed auditory hallucinations involving music or songs, the identified trigger drugs, the availability of detailed patient information, and publications written in English. The exclusion criteria were auditory hallucinations not involving songs and music, no suspected trigger drugs, no detailed patient information, not in English, and abstracts only. These criteria were employed to ensure the relevance, comprehensiveness, and quality of the selected literature for the review.

## 3 Results

**FIGURE 1:**
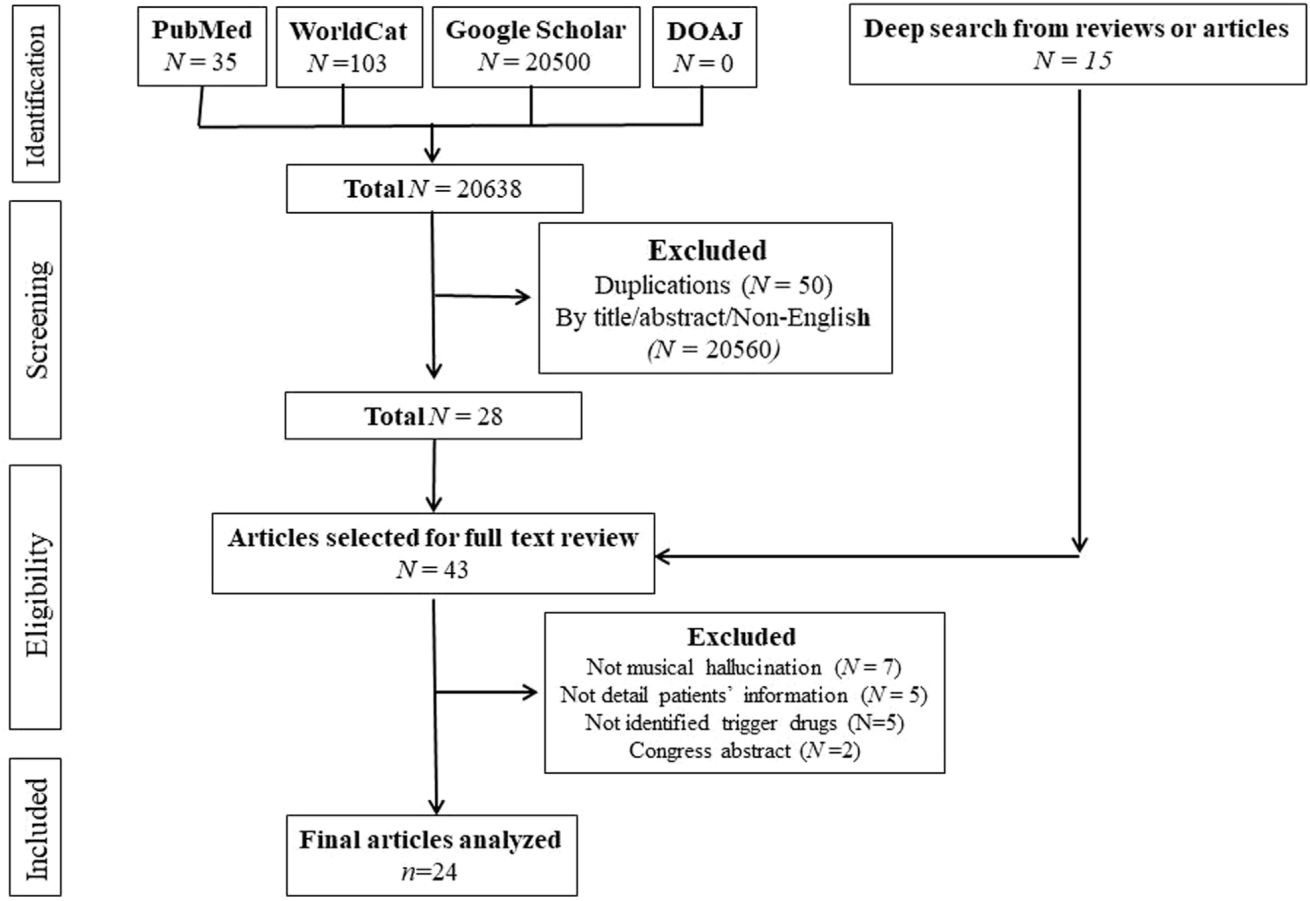
The Flowchart of Literature Search. Legends: Twenty-four articles met the inclusion criteria.

Twenty-four articles met our inclusion criteria (Vallada and Gentil, 1991; Terao, 1995; Kumagai et al., 2003; Padala et al., 2010; Muraosa et al., 2020; Kobayashi et al., 2004; Gondim Fde et al., 2010; Kataoka and Ueno, 2014; Keeley et al., 2000; Moore, 2003; Davies and Quinn, 2005; Powers et al., 2015; Agrawal and Sherman, 2004; Zonios et al., 2008; Fisman, 1991; Curtin and Redmund, 2002; al-Zahawi et al., 1988; Song and Jung, 2019; Gilbert, 1993; Tomar and Cheung, 2007; Blackman et al., 2019; Elikowski et al., 2021; Kanemura et al., 2010; Allen, 2008). The flow of the literature search is illustrated in Figure 1. These selected articles provided information on 27 patients.

### 3.1 Patients’ general information and background diseases

The analysis incorporated 27 cases documented across 24 articles. Among these cases, the mean age at the onset of the hallucinations was 58.3 years (range 21–88). Females accounted for the majority, with nineteen patients (67.9%).

Supplementary Table S1 outlines the demographic and clinical characteristics of the cohort under study. Table 1 shows background diseases and conditions. Approximately one-third of the patients (33.3%) reported a history of reduced hearing or hearing loss. Psychiatric disorders were prevalent among the cohort, with nine individuals (33.3%) presenting conditions such as bipolar disorder, anxiety, depression, panic disorder, and dissociative disorder. Cancers or leukemia were reported in seven patients (25.9%), while neurodegenerative diseases, specifically Parkinson’s and Lewy Body Dementia, were observed in three elderly individuals (11.1%). Notably three otherwise healthy individuals (11.1%) experienced musical hallucinations following ketamine intravenous infusion in a placebo-controlled experiment (Powers et al., 2015).

**TABLE 1:** Background diseases/conditions (N = 27).

Background diseases	N	%
Hypoacusis	9	33.3
Psychiatric disorders	9	33.3
Depression + Anxiety (3)		
Depression + Panic attack (1)		
Depression (2); Dysthymia (1)		
Anxiety + Bipolar Disorder (1)		
Dissociate disorder + Panic Attack (1)		
Cancer/lymphoma/leukemia	7	25.9%
Acute myeloid leukemia (1); melanoma (1)		
Non-Hodgkin’s lymphoma (2)		
Lung cancer (1)		
Bronchus cancer (1)		
Mesothelioma (1)		
Neurodegenerative disorders	3	11.1%
Parkinson (2)		
Dementia with Lewy Bodies (1)		
Chronic Otitis	2	7.4%
Osteoarthritis	2	7.4%
Chronic heart failure	1	3.7%
Chronic renal failure	1	3.7%
Transient ischemic attack	1	3.7%
Healthy volunteer	3	11.1%

Abbreviations: N, numbers.

Some patients had multiple background diseases together, which is why the total percentage is >100% in Table 1. These findings provide a comprehensive overview of the diverse clinical backgrounds associated with drug-induced musical hallucinations in the examined cohort.

### 3.2 Triggering drugs

Table 2 delineates the identified trigger drugs in 27 cases suspected of inducing musical hallucinations. Among these cases, 18.5% were associated with antidepressants, while 11.1% were linked to each of the categories: anti-Parkinson drugs, opioids, ketamine, and voriconazole. Benzodiazepines, ceftazidime, and vasodilators accounted for a total of 22.2% (each 7.4%). One instance (3.7%) was attributed to alcohol withdrawal. The remaining 11.1% involved singular triggers, namely, amiodarone, corticosteroids, and aspirin.

**TABLE 2:** Trigger drugs to musical hallucination (N = 27).

Drug category	Drugs	N	Total numbers (%)
Antidepressants	Paroxetine	2	5 (18.5)
	Mirtazapine	1	
	Imipramine	1	
	Clomipramine	1	
Antiparkinson	Amantadine	1	3 (11.1)
	Pramipexole	1	
	Bromocriptine	1	
Opioids	Tramadol	1	3 (11.1)
	Oxycodone	1	
	Morphine	1	
NMDA antagonist	Ketamine	3	3 (11.1)
Antifungal	Voriconazole	3	3 (11.1)
Benzodiazepines	Lorazepam/Temazepam	1	2 (7.4)
	Lormetazepam	1	
Antibiotics	Ceftazidine	2	2 (7.4)
Vasodilators	Dipyridamole	1	2 (7.4)
	Pentoxifylling	1	
Alcohol	Alcohol withdrawal	1	1 (3.7)
Anti-arrhythmic	Amiodarone	1	1 (3.7)
Corticosteroid	Betamethasone	1	1 (3.7)
Non-steroid anti-inflammatory drugs	Aspirin	1	1 (3.7)

Abbreviation: N, numbers; NMDA, N-methyl-D-aspartate receptor.

### 3.3 Initiation of musical hallucination

Nineteen patients provided details regarding the onset time of musical hallucinations following the administration of trigger drugs, ranging from less than 75 min to 240 days. The mean initiation time was 21.4 days, with a median of 2 days. A considerable proportion of patients, sixteen (59.3%), noted the onset occurring in less than 10 days after the administration of the trigger drug.

Beyond musical hallucinations, six patients simultaneously encountered visual hallucinations, and one patient reported disssociation. These additional manifestations further contribute to the diverse perceptual experiences associated with possible drug-induced side effects in the examined cohort.

### 3.4 Description of musical hallucination

As illustrated in Table 3, the characteristics of the music or songs experienced during musical hallucinations exhibited a diverse range. Patients showed familiarity with the majority of the songs from their youth, although some songs were novel to them. The repertoire included various genres, including holiday songs, church hymns, and patriotic melodies. This heterogeneity underscores the broad spectrum of musical content encountered in the experiences of musical hallucinations within the studied cohort.

**TABLE 3:** Features of musical hallucination.

Category of drugs	Trigger drugs’ names	Characters of musical hallucinations
Antidepressants	Mirtazapine	Elevator music
	Imipramine	Nursery rhymes and supporting songs
	Clomipramine	Familiar songs: the national anthem, hallelujahs, and other choral pieces; sometimes the patient could select the music at will
	Paroxetine	An opera song; background music from a video game; a recorder melody played by a strange face; undefined music
Antiparkinson agents	Amantadine	Patriotic or lyrical songs, Brazilian National Anthem, Silent Night, Boi-Da-Cara-Preta, Olhos Verdes
	Pramipexole	Quiet piano or songs on a loud radio or background music
	Bromocriptine	Karaoke songs
Opioids	Tramadol	“Two voices singing accompanied by an accordion and a banjo, singing songs, songs by Josef Locke--old songs”
	Oxycodone	Specific melodies such as Ave Maria, Silent Night, Jingle Bells, America the Beautiful, and Let Me Call You Sweetheart. Each was sung by a solo male voice accompanied by an orchestra
	Morphine	Classical music that had been a favorite piece of her late husband
Benzodiazepines	Lorazepam and temazepam	Popular songs and hymns including “Waltzing Matilda”, “The Yellow Rose of Texas”, “Rock of Ages”, and “Happy Birthday”
	Lormetazepam	Children’s songs
NMDA antagonist	Ketamine	“You’ll Never Walk Alone”; drums music (unfamiliar); songs from the Nutcracker, the Beatles, and David Bowie
Antifungal	Voriconazole	Christmas songs (familiar and unfamiliar), Do You Hear What I Hear, party songs
Antibiotics	Ceftazidime	Undefined
Antiplatelets/Vasodilators	Dipyridamole	Church choir songs from childhood
	Pentoxifylline	Church choir songs from childhood
Alcohol withdrawal	Alcohol withdrawal	Psychedelic rock song; voices reading a poem in a rhyming manner
Antiarrhythmic	Amiodarone	Folk songs
Steroid	Betamethasone	Opera/piano concert music
NSAID	Aspirin	Big band songs from the 1930s and 1940s that were popular during the patient’s youth, such as “When Irish Eyes Are Smiling”

Abbreviations: NSAID, non-steroidal anti-inflammatory drugs.

### 3.5 Electroencephalography and brain images

Results from electroencephalography (EEG) were available for 9 (33.3%) patients, all of which were within the normal range (Fisman, 1991; Vallada and Gentil, 1991; Gilbert, 1993; Curtin and Redmund, 2002; Kumagai et al., 2003; Agrawal and Sherman, 2004; Allen, 2008; Gondim Fde et al., 2010; Powers et al., 2015). EEG findings excluded active seizures in those nine patients.

Computed tomography (CT) scans were performed on 6 (22.2%) patients (Fisman, 1991; Vallada and Gentil, 1991; Tomar and Cheung, 2007; Gondim Fde et al., 2010; Powers et al., 2015; Song and Jung, 2019). Five patients yielded either normal or slight atrophy consistent with normal aging (Vallada and Gentil, 1991; Tomar and Cheung, 2007; Gondim Fde et al., 2010; Powers et al., 2015; Song and Jung, 2019). However, one patient exhibited an abnormal CT scan, revealing “slight prominence of the left temporal horn, and the right lateral ventricle was minimally larger than the left” (Fisman, 1991).

Magnetic resonance imaging (MRI) was conducted on 3 (11.1%) patients (Gilbert, 1993; Kobayashi et al., 2004; Kataoka and Ueno, 2014), with only one patient displaying abnormal results (Kobayashi et al., 2004). The abnormal MRI findings included “generalized cortical atrophy with several small lesions in the deep white matter of the cerebral hemispheres” (Kobayashi et al., 2004).

These diagnostic images excluded brain tumors, and acute ischemic or hemorrhagic strokes.

### 3.6 Treatments and outcomes of musical hallucinations

As shown in Table 4, in response to the occurrence of musical hallucinations, various interventions were implemented: 1) The trigger drugs were terminated; 2) The dosage of trigger drugs was reduced; 3) The administration route was altered from intravenous (IV) to oral; 4) Extended-release pramipexole was switched back to immediate-release pramipexole in one case; 5) The trigger drugs were changed to another corticosteroid, sedative, or anti-Parkinson drug; 6) Besides termination of trigger drugs, sedatives, tricyclic antidepressants, or atypical antipsychotics were added; 7) Benzodiazepines were used to treat alcohol withdrawal in one case.

**TABLE 4:** Treatments and outcomes of musical hallucinations (N = 27).

Strategy	Musical hallucinations disappeared	Musical hallucinations reduced but continued
Termination of trigger drug only	11 (40.7%)	1 (3.7%)
Dose reduction	4 (14.8%)	1 (3.7%)
Termination of trigger drug + TCA or BZD or atypical antipsychotics	3 (11.1%)	1 (3.7%)
Switching to oral route; switching back to immediate release formula	2 (7.4%)	0
Switching to other sedative, or other corticosteroid	2 (7.4%)	0
Switching from bromocriptine to biperiden^a^	1 (3.7%)	0
Adding BZD (alcohol withdrawal)	1 (3.7%)	0
Total	24 (88.9%)	3 (11.1%)

^a^ Biperiden (anti-muscarinic) and bromocriptine are drugs to treat Parkinson’s disease or syndrome.

Abbreviations: BZD, benzodiazepines; TCA, tricyclic antidepressants.

Twenty-four (88.9%) patients reported complete resolution of hallucination after the initiation of the above treatments. If the alcohol withdrawal case is not counted, then 23/27 (85.2%) fully recovered after modulating trigger drugs with or without additional pharmacotherapy. Among these 24 patients, only 21 patients specified when the musical hallucination stopped. The therapy for musical hallucination was effective within an average of 5.6 days (range 75 min–21 days). The total duration of musical hallucination in those 21 patients extended from 75 min to 23 days, with a mean duration of 8.7 days. Three patients whose musical hallucinations disappeared did not define the time.

As shown in Table 4, more than half of musical hallucinations completely disappeared by simple termination of trigger drugs (40.7%) and dose reduction (14.8%). Changing administration routes or formulas or switching to similar drugs resulted in further musical hallucination resolution (14.8%). If musical hallucinations persisted after stopping the trigger drugs, additional medications, such as risperidone, clonazepam, sultopride, and amitriptyline, led to symptom resolution (11.1%). A benzodiazepine (clonazepam) successfully controlled the musical hallucinations in a patient suffering from alcohol withdrawal (3.4%).

In the only case of aspirin-induced musical hallucination, the patient was an elderly female with hearing loss, wore bilateral hearing aids, and took a high dose (12 tablets of 300 mg) of aspirin daily for arthritis with a blood salicylate level higher than the therapeutic range (Allen, 2008). When the aspirin dose was reduced by half, her musical hallucination stopped a few days later (Allen, 2008). This supports that the overdose of aspirin caused musical hallucinations in this case.

Unfortunately, musical hallucinations were reduced but continued in 3 patients (11.1%) for as long as 1 year. Those three patients all had background diseases: depression + anxiety (1 patient) (Curtin and Redmund, 2002); depression + anxiety + panic attack + reduced hearing (1 patient) (Vallada and Gentil, 1991); bilateral hearing loss + chronic otitis media (1 patient) (Song and Jung, 2019).

These findings provide insights into the varied temporal aspects and outcomes associated with drug-induced musical hallucinations within the analyzed cases.

## 4 Discussion

### 4.1 Etiology

**FIGURE 2:**
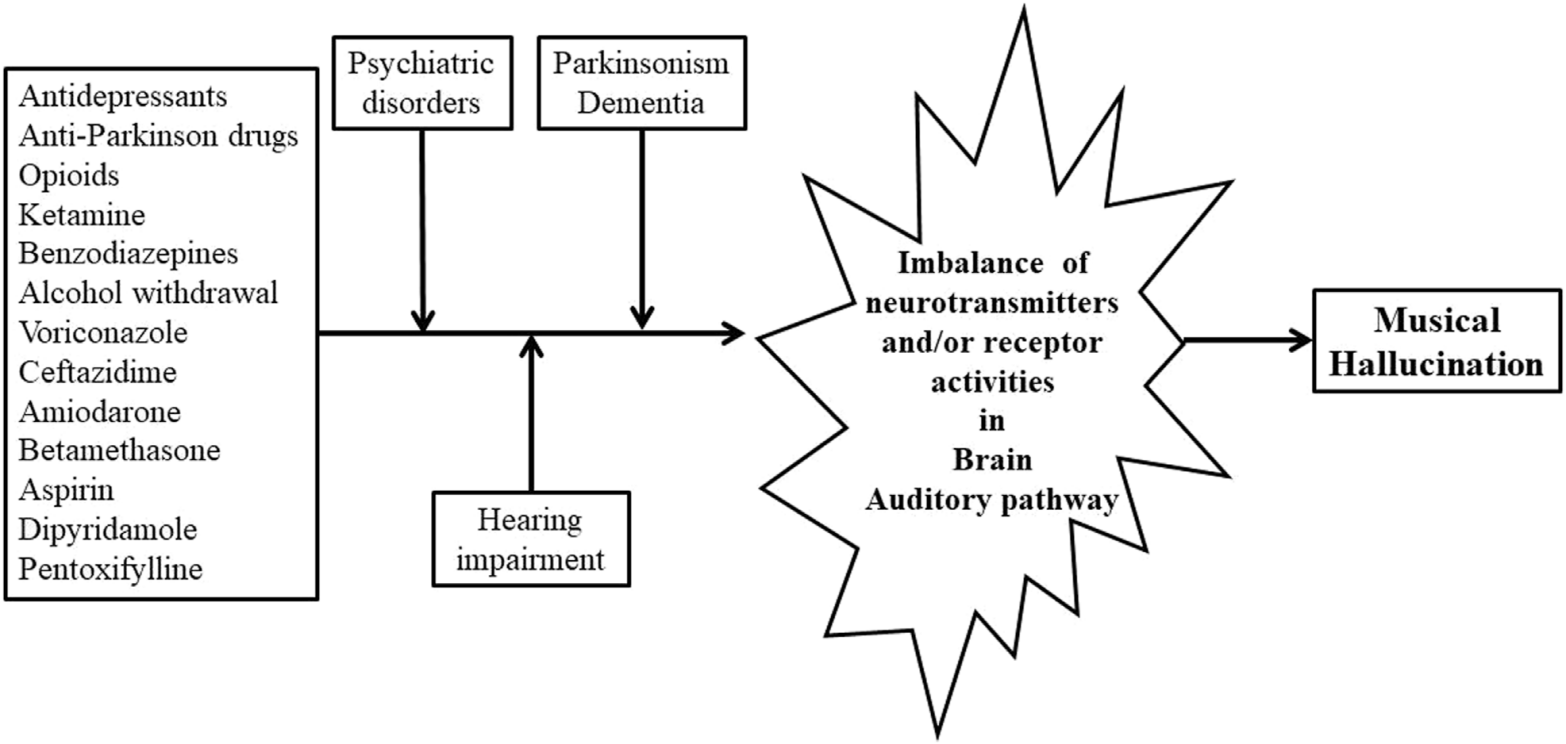
Hypothesized Etiology for Drug-Induced Musical Hallucination. Legends: The background diseases encompass hearing loss, psychiatric disorders, dementia, and Parkinson’s, all of which are associated with an imbalance of neurotransmitters or receptor activities. Trigger drugs exacerbate this imbalance in the brain on top of the background diseases, inducing musical hallucinations. Abbreviation: GABA = γ-Aminobutyric acid.

Among our case series focusing on drug-induced musical hallucinations, 33.3% of patients exhibited hearing loss or impairment, 33.3% had mental disorders, 25.9% presented with cancers, 11.1% with neurodegenerative disorders, and 7.4% with chronic otitis. Some patients had multiple medical conditions concurrently. Many drugs were suspected as trigger drugs, such as antidepressants, anti-Parkinson’s, opioids, etc. The exact pathophysiology remains elusive, however, we propose a multifactorial model with an imbalance of neurotransmitters/receptor activities contributing to the development of drug-induced musical hallucinations, as shown in Figure 2.

### 4.2 Imbalance of neurotransmitters/receptor activities

#### 4.2.1 Trigger drugs

The identification of drug-induced musical hallucinations relied on the exclusion of other potential causes and examination of the temporal relationship between drug use and the onset of musical hallucinations. The current study highlights six prominent drug categories associated with musical hallucinations: antidepressants, antiparkinson drugs, opioids, voriconazole, ketamine, and benzodiazepines. Except for voriconazole, each of these can modulate neurotransmitter levels or receptor functions in the brain. Antidepressants can enhance norepinephrine, serotonin, or dopamine functions, while antiparkinsonian drugs predominantly elevate dopamine levels or activate dopamine receptors. Opioids decrease various neurotransmitters but increase dopamine in the nucleus accumbens by disinhibition (Tanda et al., 1997). Benzodiazepines augment γ-Aminobutyric acid (GABA)-mediated inhibition in the central nervous system. Ketamine, an N-Methyl-D-aspartate receptor antagonist akin to phencyclidine, is known for inducing dissociation and hallucinatory effects. Alcohol, like barbiturates, enhances GABA-mediated inhibition, therefore, alcohol withdrawal may lead to rebound central nervous system excitation, potentially resulting in hallucinations, including musical ones.

Voriconazole can enter the brain, with concentrations in brain tissue exceeding 50% of plasma concentration (Agrawal and Sherman, 2004). While voriconazole is acknowledged for inducing visual hallucinations in up to 32% of patients (Jansen et al., 2017), instances of voriconazole-induced musical hallucinations are infrequent, and the underlying mechanisms remain unknown.

Aspirin is found to inhibit GABA transmission and stimulate the auditory pathway (Stolzberg et al., 2012). This may contribute to aspirin overdose-induced tinnitus, which is a simple auditory hallucination. Hence, aspirin may provide enough stimulus to the auditory cortex of the brain resulting in complex auditory hallucinations, such as musical hallucinations (Allen, 2008).

Ceftazidime, amiodarone, dipyridamole, pentoxifylline, and betamethasone possess sufficient lipid solubility to enter the central nervous system, although their mechanisms inducing musical hallucinations remain unclear. Dipyridamole inhibits platelet aggregation, and dipyridamole and pentoxifylline exhibit vasodilatory functions in both the central nervous system and peripheral arteries.

We hypothesized that trigger drugs or alcohol withdrawal may disrupt the balance of neurotransmitters or their receptor activities in the brain, particularly in the auditory pathway of the temporal lobes, leading to the manifestation of musical hallucinations.

#### 4.2.2 Psychiatric disorders

In this study, psychiatric disorders were observed in 33.3% of patients, including conditions such as depression, anxiety, bipolar disorder, and panic attacks. Similarly, in another study involving 393 patients with musical hallucinations, 39% had psychiatric disorders (Golden and Josephs, 2015). It is noteworthy that even in the absence of trigger drugs, musical hallucinations have been reported as idiopathic in individuals over 65 with depression (Pasquini and Cole, 1997; Cope and Baguley, 2009). Although the exact pathophysiology of psychiatric disorders is unclear, an imbalance of neurotransmitters or their receptor activities in the brain has been hypothesized as one of the contributing factors. For instance, decreased serotonin, dopamine, or norepinephrine may lead to depression, and medications that increase these neurotransmitters have shown success in treating depressive symptoms.

#### 4.2.3 Neurodegenerative disorders

The current study encompasses two cases of Parkinson’s disease and one case of Lewy body disease (11.1%). This is consistent with what other investigators found. Golden and Josephs identified neurodegenerative disorders as a significant risk factor in musical hallucinations, with 16.5% in a 393-patient cohort, including Lewy body dementia (14/393, 3.6%), Alzheimer’s dementia (18/393, 4.6%), unspecified dementia (22/393, 5.6%), and Parkinson disease (9/393, 2.3%) (Golden and Josephs, 2015). Dopamine reduction in the substantia nigra contributes to Parkinson’s disease, while individuals with Alzheimer’s or Lewy body dementia exhibit decreased acetylcholinergic neurons. This neurotransmitter or receptor dysregulation may lower the threshold for spontaneous activity in the auditory association cortex.

#### 4.2.4 Hearing impairment

In the current study, 33.3% of patients demonstrated varying degrees of hearing impairment. It is essential to highlight that, although musical hallucinations can be linked solely to isolated peripheral hearing dysfunction, hearing impairment is not a necessary condition for the occurrence of musical hallucinations (Golden and Josephs, 2015). Most individuals with deafness do not report instances of musical hallucinations. The hypothesis suggests that hearing impairment may eliminate inhibition in the cortex associated with hearing, resulting in an amplification of sound recognition (Griffiths, 2000).

#### 4.2.5 Other conditions

In this study, seven cases featured patients with peripheral cancers, wherein voriconazole, ceftazidime, and betamethasone were employed. The immunity of patients with peripheral cancers is reduced, and antimicrobials are most likely applied to prevent or treat infections. How the conditions of peripheral cancers affect central perception is unknown. Although the mechanism of drug-induced musical hallucinations remains unclear, it is highly suggested that trigger drugs are more likely than cancers to contribute to musical hallucinations.

#### 4.2.6 The hypothesis of mechanism of drug-induced musical hallucination

In cases where patients have an underlying disorder capable of directly causing musical hallucinations, the introduction of trigger drugs may precipitate their development by further exacerbating the imbalance of neurotransmitters or their receptor activities in the brain, as illustrated in Figure 2. Supporting this hypothesis, musical hallucinations ceased in 23/27 (85.2%) of patients following basic modulations on trigger drugs in our case series. It is difficult to pinpoint single neurotransmitter/pathway currently, future research is warranted to find out disrupting either one neurotransmitter/pathway (serotonin, dopamine, or GABA) or a combination of nerotransimtters/pathways in the auditory cortex of the brain could lead to drugs-inducing musical hallucination.

### 4.3 Diagnosis and treatment of drug-induced musical hallucination

The diagnosis relies on the exclusion of alternative causes. Given that structural changes in the brain, such as tumors, ischemic strokes, and hemorrhages, account for 9% of patients with musical hallucinations (Golden and Josephs, 2015), brain imaging, including CT or MRI scans, is recommended. It is essential to rule out brain infections as well. Once structural changes or infections are excluded, suspicion of drug-induced musical hallucination arises when there is a temporal correlation between the onset of musical hallucination and alterations in the treatment regimen for the underlying disease.

The cessation or modification of trigger drugs, through dose reduction, switching to different routes or formulas, or switching to similar drugs, typically results in the resolution of musical hallucinations in most patients in less than 1 month. If the symptoms persist, atypical antipsychotics, tricyclic antidepressants, and benzodiazepines have proven effective. The rapid resolution of musical hallucinations after those treatments further supports the diagnosis of drug-induced musical hallucinations.

In our case series, three patients’ musical hallucinations continued for 6 months or longer than 1 year after the above-mentioned therapy. Those three patients had hearing loss, ottis, or psychiatric disorders. Because of the ineffectiveness of modulating the suspected trigger drugs, the diagnosis of drug-induced musical hallucination is in doubt. In these three patients, the underlying diseases may contribute dominantly to the musical hallucination occurrence and persistence.

### 4.4 Strengths and limitations

To the best of our knowledge, the current study represents the initial comprehensive review of drug-induced musical hallucinations. However, there are inherent limitations to be acknowledged. As highlighted by Coebergh et al. (2015), none of the suspected trigger drugs have been investigated with placebo-controlled trials for musical halucination, raising concerns about the reliability of predicting the causative effects of trigger drugs. The absence of placebo-controlled information across all articles limits the robustness of the findings in our case series. Additionally, a low incidence rate of drug-induced musical hallucinations poses challenges in calculating prevalence, while the observational nature of the studies inhibits the generation of correlations. The inclusion criterion of articles with detailed patient information may introduce a potential bias, excluding other trigger drugs that were not published or lacked detailed information. Furthermore, the absence of experimental evidence supporting our hypothesis on the mechanism of drug-induced musical hallucination underscores the need for further research in this area.

### 4.5 Conclusion

In summary, the primary suspected trigger drugs for inducing musical hallucinations encompass antidepressants, antiparkinson drugs, opioids, ketamine, voriconazole, ceftazidime, and benzodiazepines. The diagnostic approach depends on exclusion. Patients with underlying conditions such as hearing impairment, psychiatric disorders, neurodegenerative diseases, or cancers exhibit a heightened susceptibility to drug-induced musical hallucinations. Imbalances in neurotransmitters or their receptor activities induced by trigger drugs are posited as contributing factors. Treatment strategies involve discontinuing or modifying trigger drugs, with or without additional pharmacotherapy. There is a pressing need for further research to identify possible trigger drugs and to delve into the underlying mechanism of drug-induced musical hallucinations.
