# Supplementary material for: Drug-induced musical hallucination
Source: Front Pharmacol. 2024 May 22;15:1401237. doi: 10.3389/fphar.2024.1401237 (PMC11150696; doi:10.3389/fphar.2024.1401237)
Supplement: Supplementary file 1 [file Table1.pdf]

Supplemental Table

S1 Table. Patients' General Information (N=27)

**Reference**	**Age**	**Sex**	**Diseases**	**Concomitant medications**	**Trigger drugs**	**Initiation of MH after trigger (days)**	**Other hallucination/ psychiatric**	**Treatment of MH**	**Disappear MH after treatment of MH (days)**	**Duration of MH (days)**
Vallada, et al., 1991	67	F	Anxiety, depression, panic, decreased hearing		Clomipramine in increasing dosage up to 50 mg/day x 2w	2		The dose of clomipramine was reduced to 10 mg	Decreased	365 (continued)
Terao, 1995	40	M	Dysthymia	Lithium, imipramine, levomepromazine, flunitrazepam	Imipramine dosage was further increased to 150 mg a day	7		The imipramine dose was reduced to 50 mg	Disappeared (undefined time)	
Kumagai, et al., 2003	28	F	Dissociative disorder, panic attack		Paroxetine	30	Visual	Termination of paroxetine; sultopride (Atypical antipsychotic) added	16	16
Padala, et al., 2010	88	M	Depression	Trazodone	Mirtazapine 45mg	3		The dose of mirtazapine was reduced to 30 mg	1	1
Muraosa, et al., 2020	22	F	Panic attack, depression	Olanzapine, lorazepam	Paroxetine	10		Termination of paroxetine on day 31	2	23
Kobayashi, et al., 2004	67	M	Dementia with Lewy bodies	Donepezil, quetiapine, triazolam, tamsulosin,	Bromocriptine	Undefined		Termination of bromocriptine; biperiden (Anti-M) added	21	21
Gondim, et al., 2010	77	F	Early Parkinson; presbyacusis-hearing aid	Levodppa, benserazide	Amantadine			Termination of amantadine on day 2	3	3
Kataoka & Ueno, 2014	72	F	Parkinson's Disease	Levodopa, Entacapone, Zonisamide, Selegiline	Pramipexole's immediate release was changed to an extended release	120		Switched back to immediate release pramipexole	12	12
Keeley, et al., 2000	74	M	Lung cancer		Tramadol	soon (undefined)		Termination of tramadol	2	2

Moore, 2003	80	F	Osteoarthritis, renal failure, deaf		Oxycodone, an extra dose	Undefined		Termination of oxycodone	14	17
Davies & Quinn, 2005	74	F	Bronchus cancer; COPD; left deafness		Morphine	2		Termination of morphine	1	1
Powers, et al., 2015	21	F	Healthy volunteer		Low dose ketamine IV infusion 75 min	0.05	Visual	Termination of ketamine at 75min	0.05	0.05
	21	F	Healthy volunteer		as above protocol	0.05	Dissociation	Termination of ketamine at 75min	0.05	0.05
	30	M	Healthy volunteer		as above protocol	0.05	Visual	Termination of ketamine at 75min	0.05	0.05
Agrawal & Sherman, 2004	78	M	AML; prophylaxis for fungi	Verapamil	Voriconazole	1		Termination of voriconazole	3	8
Zonios, et al., 2008	62	M	Melanoma		Voriconazole IV	1	Visual	Termination of voriconazole on day 5	1	5
	50	F	NHL, Allo HSCT, GVHD		Voriconazole IV	1	Visual	Switched to oral voriconazole on day 4	1	5
Fisman 1991	65	F			Lorazepam /temazepam	240		Termination of lorazepam and temazepam; switched to chloral hydrate	Disappeared but undefined time	
Curtin & Redmund, 2002	45	F	Depression, anxiety		Lormetazepam	a few days		Termination of lormetazepam; amitriptyline added	Reduced	240 (continued)

al-Zahawi, et al., 1988	62	F	Non-Hodgkin's lymphoma of 12 years, recurrent lung infection		Ceftazidime	1	Visual	The ceftazidime dose was reduced by 50%	2	4
Song & Jung, 2019	51	F	Chronic otitis media bilaterally, hearing loss		Ceftazidime	1		Termination of ceftazidime at 2 weeks	Decreased	180 (continued)
Gilbert, 1993	88	F	Chronic otitis media bilaterally, deaf	Amitriptyline was added because of MH for sleeping	Pentoxifylline	a few days		Termination of pentoxifylline and amitryptiline; clonazepam added	7	21
Tomar & Cheung, 2007	83	F	TIA, Deaf		Dipyridamole	?		Termination of dipyridamole 3 weeks later; risperidone added	21	21
Blackman, et al., 2019	25	M	Alcoholism, depression		Alcohol stopped 2 days ago	2		Lorazepam	3	3
Elikowski, et al., 2021	77	M	Advanced heart failure, hypoacusis		Amiodarone (overdose	1		Termination of amiodarone on day 3	1	3
Kanemura, et al., 2010	57	F	Malignant pleural mesothelioma, anxiety, bipolar	Fentanyl patch	Betamethasone	2		Termination of betamethasone; changed to prednisone on day 11	7	16
Allen, 2008	70	F	bilateral hearing aids use; arthritis		12 aspirin tablets (300 mg x12) per day	?		The dose of aspirin was reduced by 50% to 300mgx 6 per day	A few days	

Abbreviations: Allo HSCT = Allogeneic Hematopoietic Stem Cell Transplant; AML = Acute myeloid leukemia; COPD = Chronic obstructive pulmonary disease; GVHD = Graft-versus-host disease; MH = Musical hallucination; NHL = *Non-Hodgkin's lymphoma*; TIA = Transient ischemic attack;
